# Association of Hepatic Steatosis With Coronary Artery Disease by Studying the Role of Individual and Demographic Risk Factors

**DOI:** 10.7759/cureus.29444

**Published:** 2022-09-22

**Authors:** Omar Alserihy, Yasser Alsallumi, Fahad Alzahrani, Abrar S Al-Sulami

**Affiliations:** 1 Radiology, King Abdullah Medical City, Makkah, SAU; 2 Microbiology, Taif University, Taif, SAU

**Keywords:** hepatic steatosis, non-alcoholic fatty liver disease (nafld), metabolic syndrome (mets), non-alcoholic steatohepatitis (nash), atherosclerosis, fatty liver disease, fatty liver, coronary artery disease, computed tomography, calcium score

## Abstract

Background

In this study, we aimed to explore the possible association between hepatic steatosis (HS) and coronary artery disease (CAD) by calculating the patients’ calcium scores and comparing clinical and laboratory parameters of patients in King Abdullah Medical City (KAMC), Makkah, Kingdom of Saudi Arabia (KSA). The role of risk factors associated with HS was also assessed.

Methodology

The medical records of 79 patients who underwent coronary cardiac computed tomography (CT) for calcium scoring and enhanced or non-enhanced CT scans of the abdomen and pelvis at the Department of Radiology, KAMC, Makkah, KSA, between April 2012 and April 2013 were collected and analyzed.

Results

The overall prevalence of HS was 32.9%. Gender, age, and body mass index were significantly associated with HS. Low-dose unenhanced CT is a promising screening test for the determination of HS. A severe grade of calcium score was significantly associated with HS, while hypertension had no significant relation with HS. Biomarkers such as blood urea nitrogen, creatinine, cholesterol, and triglycerides had a significant association with HS, while other liver function tests and lipid profile values did not have a significant association. Bilirubin was significantly higher in non-fatty liver than in fatty liver. Furthermore, higher grades of calcium score were significantly associated with fatty liver in non-hypertensive and non-diabetic patients.

Conclusions

CAD is closely associated with HS. Moreover, diabetes mellitus and hypertension play a critical role in the development of HS.

## Introduction

Fatty liver (hepatic steatosis, HS) is a reversible condition resulting from the excessive accumulation of triglycerides within cytoplasmic vesicles of hepatocytes that is correlated with various clinical disorders [1,2]. Even though it is normal for the liver to contain some amount of fat, fatty liver is diagnosed if fat exceeds 5-10% of the total weight of the liver [3]. Fatty liver disease (FLD) has become a global health concern in children as well as adults. It develops in those who consume superfluous alcohol and in obese individuals irrespective of the influence of insulin resistance. It is also noted in various genetic and metabolic conditions which affect fatty acid metabolism [4]. When utilization of energy outpaces the calorie combustion, the unburnt energy is preserved as fat (triacylglycerol, TG) in adipose tissue which leads to obesity [5]. Obesity-linked insulin resistance represents a pathogenic event culpable for metabolic syndrome (MS) amounting to type 2 diabetes mellitus, atherosclerosis, hypertension, dyslipidemia, and HS proceeding to FLD [6]. Previously, excessive consumption of alcohol was thought to account for most FLD cases, but, recently, non-alcoholic causes of FLD have attracted noticeable attention. To bring the arising non-alcoholic causes of FLD, particularly obesity-related FLD, into attention and to differentiate this from the known alcoholic liver disease, FLD is clinically classified into two main categories, namely, alcoholic fatty liver disease (AFLD) and non-alcoholic fatty liver disease (NAFLD) [7].

HS is a progressively prevailing condition in Western countries probably due to a high-fat diet [8]. HS is detected in around 20-35% of the general adult population in the United States, with nearly 10% of these cases progressing toward NAFLD [5]. On the contrary, the incidence of steatosis in obese persons is about 75%; however, approximately 35% or more of these cases with no association with excessive alcohol consumption evolve into NAFLD [4-7]. The pertinent histological features that establish the basis of FLD include HS and steatohepatitis with advancement to cirrhosis. Both AFLD and NAFLD normally commence as HS, and upon the persistence of the cause, steatosis constantly proceeds to steatohepatitis, cirrhosis, and liver cancer [5-7]. Morphologically, HS manifests as the aggregation of small (microvesicular) or large (macrovesicular) intracytoplasmic fat droplets in the liver parenchymal cells. It is largely macrovesicular in alcoholic, diabetic, and obese individuals, as well as in certain malnutrition conditions such as acquired immune deﬁciency syndrome and kwashiorkor. In the macrovesicular type of HS, hepatocytes contain a large and single vacuole of fat that ﬁlls the cytoplasm and moves the nucleus to the outer edge giving rise to a characteristic signet ring appearance. In microvesicular HS, hepatocytes are populated by abundant small fat droplets which do not displace the centrally located nucleus to the periphery. In AFLD and long-established NAFLD, HS is mostly macrovesicular but may be admixed with microvesicular droplets in some cases [7]. In AFLD and NAFLD, the pathogenesis of the fatty change seems multifactorial. In AFLD, inhibition or impairment of peroxisome proliferator-activated receptor (PPAR)-α and PPAR-γ function and stimulation of sterol regulatory element-binding protein (SREBP) 1, the receptor molecules that control the enzymes responsible for the oxidation and synthesis of fatty acids, respectively, appear to contribute to the general fat load in the liver [9,10].

Although it is difficult to evaluate the relative roles of different factors in the development of HS, the clear result may be that increasingly steatotic hepatocytes begin to rupture or die. Apoptotic or ruptured hepatocytes release TGs, which in association with the unmetabolized very long-chain fatty acids augment the liver injury. In NAFLD, HS is associated with insulin resistance but there is increasing evidence that non-alcoholic steatohepatitis (NASH) can occur in the absence of apparent insulin resistance [9,10]. These observations propose that in NAFLD HS may occur as a simple overstorage of unmetabolized energy in hepatocytes in individuals consuming excessive energy which exceeds the energy combustion capability of PPAR-α-mediated fatty acid oxidation systems [11]. The prevalence of NAFLD is around 50% in diabetic individuals, 76% in obese individuals, and 100% in those who are morbidly obese with diabetes [12]. Therefore, the fundamental cause of fat accumulation in AFLD and NAFLD is mainly due to fatty acid synthesis and inhibition of fatty acid oxidation. HS is considered harmless in its amiable form, reversible, and, to a great extent, non-progressive if there is removal or cessation of the fundamental cause [5-7].

In addition to the known risks of growing liver injury, many studies have revealed a correlation between HS and MS. Several clinical risk factors associated with HS have been identified, particularly alcohol overuse, diabetes, dyslipidemia, insulin resistance, hepatitis, hypertension, and obesity, which are together called MS [5,13]. HS has been linked to visceral adiposity, high serum triglycerides, low serum high-density lipoproteins, and pro-inflammatory biomarkers such as C-reactive protein. It has been shown to be correlated with an expanded risk of cardiovascular events independent of variables in individuals with diabetes [14,15]. However, there is contradictory evidence of an independent association between HS and characteristics of subclinical atherosclerosis, a cardiovascular disease [16]. These findings incur more investigation as to whether HS itself intermediates the development of cardiovascular disease and if it could be included in predictive models [17].

Cardiovascular diseases include hypertension, coronary artery disease (CAD), generalized atherosclerosis, heart rate variability, myocardial infarction, sudden cardiac death, carotid artery disease, cerebrovascular accidents (stroke), and cardiac arrhythmias. CAD is the leading cause of mortality worldwide. The etiology of CAD involving atherosclerosis is well-studied [18]. CAD is also linked to various metabolic disorders, escalating concern that patients with NAFLD may also be at an increased risk of developing coronary heart disease [19]. HS can be mainly diagnosed by computed tomography (CT), nuclear magnetic resonance (NMR), ultrasound (US), and liver biopsy, while CAD can be diagnosed by angiography and CT [20]. Increasing evidence suggests that the presence of abnormal aortic, carotid, and coronary calcium deposits, as measured by CT, can be an important subclinical predictor of cardiovascular diseases such as CAD [17]. The screening benefits for arterial calcium deposition have been supposed to assist in risk stratification for coronary disease [21]. Hence, an evaluation of associations between HS and vascular calcium provides a peculiar opportunity to evaluate the role of HS as a participant in the creative pathway for atherosclerosis which leads to CAD [22].

Much research has been done to determine the relationship between NAFLD or HS and cardiovascular diseases but there are only a few studies analyzing the association between HS and CAD in the Kingdom of Saudi Arabia (KSA). The alarming increase in the prevalence of CAD indicates that more research is needed to determine the prevalence, diagnosis, causes and treatment of CAD, and its relationship with other metabolic disorders. In this study, we have attempted to expand and replicate the observations of previous studies. This cross-sectional retrospective study aimed to explore the possible association between HS and CAD by calculating patients’ calcium scores and comparing their clinical and laboratory parameters. We further examined the potential role of risk factors, namely, diabetes mellitus and hypertension in the development of HS.

## Materials and methods

This cross-sectional retrospective study was carried out at the Department of Radiology, King Abdullah Medical City (KAMC), Makkah, KSA. The initially estimated population was 224 patients who were referred to the Department of Radiology for coronary cardiac CT scans or calcium scoring and enhanced or non-enhanced CT scans of the abdomen and pelvis over a period of one year (April 2012 to April 2013). The medical records of all patients were reviewed and the data from patients’ electronic medical records were obtained. The data included clinical (age, gender, body mass index, history of diabetes mellitus, and hypertension) and laboratory (kidney function tests, liver function tests, glycemic control, lipid profile, and CT scan of coronary calcium score) parameters. Patients were identified by serial study code to protect them from social, emotional, or psychological damage. This study was carried out according to the guidelines of the Research Ethical Committee (King Abdullah Medical Research Center), Makkah, KSA. These guidelines comply with the National Committee of BioMedical Ethics guidelines, KSA, and national and international laws and policies of the National Institutes of Health, USA.

The calcium score was calculated using the Agatston score (AS), a semi-automated tool that calculates a score based on maximum density (HU) and extent of coronary artery calcification detected by an unenhanced low-dose CT scan which is routinely performed in patients undergoing cardiac CT. AS permits early risk stratification as patients with a high AS (>160) have an elevated risk for a major adverse cardiac event (MACE) [23]. The grading of CAD is based on the total calcium score (Table 1).

**Table 1:** The grading of CAD based on the total calcium score. CAD: coronary artery disease

Grade of coronary artery disease	Calcium score
No evidence	0
Minimal	1–10
Mild	11–100
Moderate	101–400
Severe	>400

Statistical analysis was done using SPSS version 16.0 (SPSS Inc., Chicago, IL, USA). Quantitative data were expressed as mean ± standard deviation (SD). Student’s t-test was used as a test of significance for quantitative data. Qualitative data were expressed as numbers and percentages (%). The significance of qualitative data was determined using the Chi-square test. In all tests, p-values of <0.05 were considered statistically significant.

## Results

In this study, the data of 79 patients were collected and a 32.9% prevalence of fatty liver was determined among the study group. Of the 79 patients, only 26 were detected with fatty liver (Table 2).

**Table 2:** Prevalence of fatty liver in the studied group.

Characteristic	Number	Percentage (%)
Non-fatty liver	53	67.1
Fatty liver	26	32.9
Total	79	100

Regarding the comparison of fatty liver (steatosis) and non-fatty liver (non-steatosis) groups in terms of age, body mass index, and gender, a significant difference (p = 0.004) was found in gender. This confirmed that females are significantly in higher association with FLD than males. The mean age for the non-fatty liver group was 58.92 ± 11.55 years, while for the fatty liver group it was 66.5 ± 10.74 years. The difference in the age between non-fatty and fatty liver patients was statistically significant (t = 2.871 and p = 0.006). The mean body mass index for the non-fatty liver group was 30.6 ± 6.14 kg/m^2^ while for the fatty liver group it was 34.65 ± 6.08 kg/m^2^. A statistically significant difference (t = 2.73 and p = 0.008) was found in the body mass index. These results indicate that old age and high body mass index are significantly associated with fatty liver (Table 3).

**Table 3:** Relation of fatty liver with some clinical parameters of the studied cohort. **: significant.

Characteristic	Individuals with fatty liver (N = 26)		Individuals with non-fatty liver (N = 53)		Test of significance
Gender: male, female	%	N	%	N	Chi-square = 8.08, p = 0.004**
	61.5, 38.5	16, 10	28.3, 71.7	15, 38	
Age, mean ± SD	66.5 ± 10.74		58.92 ± 11.55		t = 2.871, p = 0.006**
Body mass index, mean ± SD	34.65 ± 6.08		30.6 ± 6.14		t= 2.73, p = 0.008**

Quantitative data were expressed as mean and SD. Qualitative data were expressed as numbers and percentages (%). Student’s t-test was used for quantitative data and significance was considered at p-values less than 0.05. The chi-square test was used for qualitative data, and significance was considered at p-values less than 0.05.

The data for the relation of HS with CT type, hypertension, and coronary calcium score of all 79 patients were expressed as numbers and percentages. The statistically significant difference (p = 0.032) in the type of CT performed signifies the correlation of CT type with the determination level of HS. The difference in the history of hypertension between non-fatty and fatty liver patients was not statistically significant (p = 0.373). Thus, there was no significant relation between hypertension and fatty liver. Among the grades of calcium score, there was a statistically significant (p = 0.007) correlation between the severe grade of calcium and fatty liver (Table 4).

**Table 4:** Relation of fatty liver with computed tomography type, hypertension, and coronary calcium score of the studied group. Quantitative data are presented as mean and standard deviation. Qualitative data are presented as numbers and percentages. Student’s t-test is used for quantitative data. The chi-square test is used for qualitative data. **: significant.

Characteristic	Fatty liver (N = 26)		Non-fatty liver (N = 53)		Test of significance
	%	N	%	N	
CT type					
Enhanced	46.2	12	22.6	12	Chi-square = 4.55, p = 0.032**
Non-enhanced	53.8	14	77.4	41	
Hypertension					
Normotensive	57.7	15	50.9	27	Chi-square = 0.319, p = 0.373
Hypertensive	42.3	11	49.1	26	
Grade of calcium score					
No	26.9	7	26.4	14	Chi-square = 14.13, p = 0.007**
Minimal	7.7	2	9.4	5	
Mild	11.5	3	13.2	7	
Moderate	3.8	1	47.2	25	
Severe	50	13	3.8	2	

HS was correlated with some laboratory parameters (abdominal CT, kidney function tests, and glycemic control) of the patients. The study of abdominal CT showed that liver span was highly significant (t = 2.91 and p = 0.005) in non-fatty liver. In kidney function tests, the blood urea nitrogen (t = 2.86 and p = 0.007) and creatinine (t = 2.32 and p = 0.023) were significantly associated with fatty liver. Glycemic control, namely, glycogen (t = 0.124 and p = 0.84), glucose F (t = 0.132 and p = 0.89), glucose R (t = 0.225 and p = 0.82), and glycated hemoglobin (t = 0.64 and p = 0.36) were not significantly correlated with fatty liver and non-fatty liver (Table 5).

**Table 5:** Relation of fatty liver with some laboratory parameters (abdominal computed tomography, kidney function tests, and glycemic control) of the studied group. Data were expressed as mean and standard deviation (SD). Student’s t-test was used and significance was considered at p-values of less than 0.05. **: significant.

Characteristic	Fatty liver (N = 26), mean ± SD	Non-fatty liver (N = 53), mean ± SD	Test of significance
Abdominal computed tomography			
Liver	52.15 ± 29.01	70.47 ± 24.85	t = 2.91, p = 0.005**
Spleen	79.23 ± 38.67	66.48 ± 31.59	t = 1.54, p = 0.127
Kidney function tests			
Creatinine	68.15 ± 12.49	162.08 ± 36.55	t = 2.86, p = 0.007**
Blood urea nitrogen	5.64 ± 1.87	8.25 ± 7.56	t = 2.32, p = 0.023**
Glycemic control			
Glycogen	32.69 ± 4.35	32.52 ± 6.008	t = 0.124, p = 0.84
Glucose F	8.88 ± 3.12	9.9 ± 5.99	t = 0 .132, p = 0.89
Glucose R	7.91 ± 2.87	8. 2 ± 5.12	t =0.225, p = 0.82
Glycated hemoglobin	66.5 ± 10.74	58.92 ± 11.55	t = 0.64, p = 0.36

Other laboratory parameters such as liver function tests and lipid profiles of the patients in the studied cohort were also compared between the fatty and non-fatty liver individuals. In liver function tests, only total bilirubin (t = 2.77 and p = 0.007) was significantly higher in non-fatty liver, which demonstrated that it was in an inverse significant association with HS. While the rest of the liver function tests, namely, alkaline phosphatase (ALP) (t = 0.58 and p = 0.55), albumin (t = 0.871 and p = 0.38), aspartate aminotransferase (AST) (t = 0.78 and p = 0.43), alanine aminotransferase (ALT) (t = 0.78 and p = 0.43), and total protein (TP) (t = 1.021 and p = 0.31) did not have a significant correlation with HS. In the lipid profile, cholesterol (t = 2.871 and p = 0.006) and triglyceride (t = 1.99 and p = 0.036) levels were significantly higher in fatty liver than in non-fatty liver and were significantly associated with HS. Other constituents of lipid profile, namely, G-protein (t = 1.72 and p = 0.081), high-density lipoprotein (HDL) (t = 1.07 and p = 0.28) and low-density lipoprotein (LDL) (t = 0.187 and p = 0.85) were not significantly correlated with HS (Table 6).

**Table 6:** Relation of fatty liver with laboratory parameters (liver function tests and lipid profile) of the studied group. Data were expressed as mean and standard deviation (SD). Student’s t-test was used and significance was considered at p-values less than 0.05. **: significant.

Characteristic	Fatty liver (N = 26), mean ± SD	Non-fatty liver (N = 53), mean ± SD	Test of significance
Liver function tests			
Total bilirubin	8.03 ± 2.27	12.06 ± 11.55	t = 2.77, p = 0.007**
Alkaline phosphatase	84.11 ± 35.01	79.97 ± 25.53	t = 0.58, p = 0.55
Albumin	37.64 ± 7.3	39.16 ± 11.55	t = 0.871, p = 0.38
Aspartate aminotransferase	18.57 ± 5.54	20.52 ± 11.87	t = 0.78, p = 0.43
Alanine aminotransferase	19.92 ± 8.78	21.94 ± 11.60	t = 0.78, p = 0.43
Total protein	74.26 ± 5.28	73.92 ± 5.28	t = 1.021, p = 0.31
Lipid profile			
G-protein	39.07 ± 25.28	30.15 ± 19.71	t = 1.72, p = 0.081
Cholesterol	22.62 ± 7.93	4.61 ± 1.24	t = 2.871, p = 0.006**
Triglycerides	1.45 ± 1.53	1.01 ± 0.75	t = 1.99, p = 0.036*
High-density lipoprotein	0.96 ± 1.85	1.03 ± 0.31	t = 1.07, p = 0.28
Low-density lipoprotein	2.81 ± 1.32	2.86 ± 0.98	t = 0.187, p = 0.85

In this study of 79 patients, the higher grades of calcium score were significantly associated with fatty liver in normotensive (p = 0.029) and non-diabetic (p = 0.007) patients (Table 7).

**Table 7:** Association of fatty liver with calcium score in normotensive and non-diabetic individuals of the studied group. Data are expressed as numbers and percentages (%). The chi-square test was used and significance was considered at p-values less than 0.05. **: significant.

Parameter	Fatty liver: normotensive (N = 11)		Non-fatty liver: normotensive (N = 26)		Test of significance
	%	N	%	N	
Grade of calcium score					
No	27.3	3	53.8	14	Chi-square = 10.67, p = 0.029**
Minimal	0	0	7.7	2	
Mild	18.2	2	30.8	8	
Moderate	9.1	1	0	0	
Severe	45.5	5	7.7	2	
	Fatty liver: non-diabetic (N = 17)		Non-fatty liver: non-diabetic (N = 30)		
Grade of calcium score					
No	23.5	4	53.3	16	Chi-square = 12.13, p = 0.007**
Minimal	11.8	2	6.7	2	
Mild	5.9	1	20	6	
Moderate	5.9	1	10	3	
Severe	52.9	9	10	3	

## Discussion

To investigate the possible association between HS and CAD, a retrospective study was conducted on 79 patients who underwent coronary cardiac CT or calcium scoring and enhanced or non-enhanced CT scan of the abdomen and pelvis over one year at KAMC. The prevalence of HS among the studied group was found to be less than 50%. This observation is analogous to the findings of the former study where the prevalence of fatty liver was less than 50%.

In this study, a significant difference was found in gender, age, and body mass index during the comparison between fatty liver and non-fatty liver. This finding demonstrates that fatty liver disease is more common in females than males, and old age is significantly associated with fatty liver than young age. Contrary to our findings regarding gender and age, a study conducted among a United States adult population found no association between demographic characteristics such as age, sex, and ethnicity with HS [24]. The result shows that a high body mass index is significantly associated with fatty liver than a low body mass index, which indicates that the body mass index is a potential risk factor for HS. This finding is supported by a previous study conducted in the United States which reported that individual risk factors such as body mass index are highly associated with HS [24].

This study also determined and compared the relationship of HS with CT type, hypertension, and coronary calcium score. The comparison of non-enhanced and enhanced CT showed a significant difference which advocates the correlation of CT type with the determination of HS [25,26]. This finding may be helpful in selecting the method to identify HS using CT and has been supported by another study that employed low-dose non-enhanced CT as a screening tool for detecting HS [24]. CT provides fast, objective, reproducible, and non-invasive assessment, and equates well with pathologic fat content, counteracting biopsy in most cases [24-26]. No significant difference was observed between the normotensive and hypertensive patients in terms of the fatty and non-fatty liver, which is contrary to the relationship between hypertension and HS. This finding contradicts previous studies that reported clinical risk factors such as obesity, hypertension, diabetes, and insulin resistance are associated with HS [5,13]. Furthermore, it has also been reported that these clinical risk factors serve as poor diagnostic screening tools that are either unacceptably insensitive or non-specific [27]. More importantly, the result shows that severe grades of calcium score have a significant correlation with HS which indicates that coronary calcium score is a potential risk factor for HS. This finding is supported by the diabetes heart study which reported that there is a significant association between coronary calcium score and HS which is mediated by metabolic disorders [22].

Several laboratory parameters (abdominal CT, kidney function tests, and glycemic control) of the patients were studied. The results of abdominal CT showed that liver span is highly significant in non-fatty liver. This finding is consistent with a previous study reporting that liver span is highly effective in determining liver infections, hepatic tumors, and MS. Blood urea nitrogen and creatinine are significantly associated with fatty liver than non-fatty liver. This finding is supported by a study that reported the possible link between HS and chronic kidney disease (CKD). These results have highlighted that HS may be a new and added risk factor for the development and progression of CKD. The vital cardiometabolic risk factors and possible common pathophysiological mechanisms shared by HS and CKD imply that both are linked to an increased risk of incident cardiovascular disease events. Glycemic control such as glycogen, glucose, glucose R, and glycated hemoglobin are not significantly correlated with fatty liver and non-fatty liver. Contrary to our findings on glycemic control, a study reported that diabetes and insulin resistance are closely associated with HS [24].

Analysis of the liver function tests showed that only total bilirubin was significantly higher in non-fatty liver, which demonstrates that it is inversely correlated to HS. A retrospective study previously established an inverse relationship between serum bilirubin with the prevalence of HS and reported a negative relation between cardiovascular disease and serum bilirubin and serum bilirubin level and CAD [28]. Liver function tests such as ALP, albumin, AST, ALT, and TP were not significantly correlated with HS. This non-significant relation of liver function tests may be attributed to the inefficient methodology employed for the examination of these biochemical markers. Contrary to our results, several studies have redefined the role and normal ranges of liver function tests such as ALP, albumin, AST, ALT, and TP with respect to HS [29]. It has also been reported that elevated AST or ALT levels are predictive biochemical markers for the presence of NAFLD if two basic criteria are met, namely, the exclusion of alternative chronic liver diseases and the presence of features of the MS [30]. In the lipid profile, cholesterol and triglyceride levels were significantly higher in fatty liver and were significantly associated with HS. Previous studies have reported that cholesterol and triglycerides are biomarkers that are closely associated with hyperlipidemia and MS correlated with HS [22]. Other constituents of lipid profile such as G-protein, HDL, and LDL are not significantly associated with HS. It has been described that serum HDL is significantly lower in HS, and LDL and G-protein as biomarkers are not associated with the presence or absence of HS [22].

In this study, the higher grades of calcium score were significantly associated with fatty liver in normotensive and non-diabetic patients. Higher grades of calcium score were directly linked to higher coronary risk, and hypertension was closely related to HS and diabetes mellitus; however, it does not necessarily mean that normotensive and non-diabetic patients are not linked with CAD [19-22]. A diabetes heart study previously reported that there was no difference in the prevalence of hypertension, CAD, and stroke between those with and without HS [22].

There are several limitations to our study. Because it was a cross-sectional retrospective investigation and not a longitudinal evaluation of HS, the data collected should have covered all the recorded characteristics of the patients. However, the data did not include the details of all the risk factors associated with HS. The sample size was not sufficient to carry out a distinctive study.

## Conclusions

We concluded that the prevalence of HS in the patients presenting to KAMC, Jeddah is almost similar to that of other countries reported earlier. This study confirmed that there is a close association between CAD and HS. Our findings exhibit that low-dose unenhanced CT is a promising screening test for detecting cases of moderate-to-severe HS by applying a 40-HU threshold. This study also affirmed that risk factors such as body mass index, gender, age, and coronary calcium score are associated with HS. Biomarkers such as blood urea nitrogen, creatinine, bilirubin, cholesterol, and triglycerides play a definite role in determining the presence or absence of HS and cardiovascular disease. While biomarkers such as glycemic control, ALP, albumin, AST, ALT, TP, LDL, HDL, and G-protein have the least or no correlation with HS. Diabetes mellitus and hypertension play a role in the development of HS. Further longitudinal studies are required to confirm the prevalence of HS and its association with individual and demographic risk factors.
